# Traveling Light and the Tyranny of Higher Expectations

**DOI:** 10.3201/eid1501.000000

**Published:** 2009-01

**Authors:** Polyxeni Potter

**Affiliations:** Centers for Disease Control and Prevention, Atlanta, Georgia, USA

**Keywords:** Art science connection, emerging infectious diseases, art and medicine, Cameron Hayes, zoonoses, pets, Herodotus, Jonathan Swift, about the cover

**Figure:**
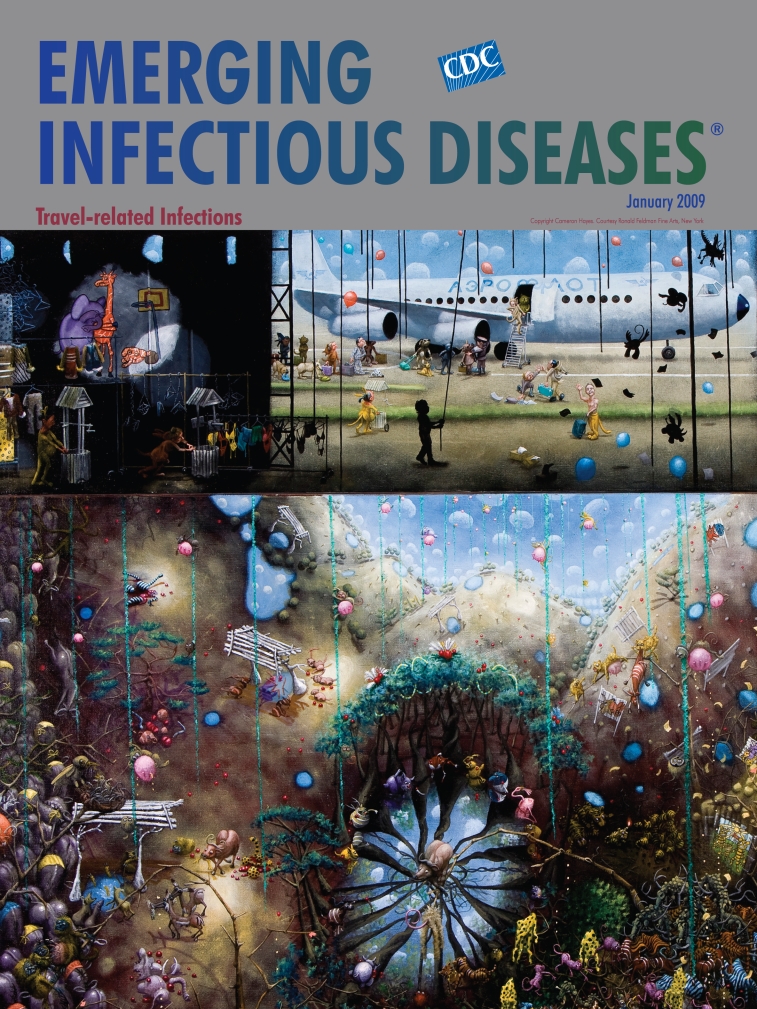
**Cameron Hayes (b. 1969) The Russians knew perfectly well that the happiness of the African animals was that they had such low expectations―before the pets were introduced (detail)** **(2008).** Oil on linen (203.2 cm × 254 cm) Copyright Cameron Hayes. Courtesy Ronald Feldman Fine Arts, New York

“Three tribes of Babylonians,” Herodotus wrote, “eat nothing but fish, which they catch and dry in the sun. They pound the dried fish in a mortar with a pestle and sift through a cloth then mix with liquid and bake like bread.” Such are their customs, he reported, “Having no physicians, they bring the sick to the agora to receive advice from passers-by who have similar ailments.”

Travel anecdotes fill Herodotus’ histories. He recorded them so that “happenings will not be lost to human memory nor great and fantastic deeds … fade.” Mocked for his accounts of outlandish behavior, Herodotus got no respect until centuries later, when similar unlikely behavior was seen elsewhere, and its anthropological and ethnographic roots were verified. Human fascination with travel to mysterious lands has occupied artists as well as writers throughout the ages. Australian painter Cameron Hayes, whose work graces this month’s cover, offers his own narrative version of travel.

Hayes, whose interests in human behavior are reflected in all his work, traces his roots far from today’s art centers, even if he exhibits in galleries all over the world. Born in Sydney and now based in Melbourne, he has explored the effects of European settlement on the Aboriginal population in Milikapiti on Melville Island off the northern coast of Australia. He has articulated in his art the loss of cultural identity and health to often well-intentioned outside influences. This journey inward sharpened his vision of today’s global scene, which he views with suspicion and satirizes without mercy in his paintings.

Hayes’ style, resistant to prevailing art trends, is narrative. His work tells a story, in the tradition of Hieronymus Bosch (c. 1450–1516), who painted fantastic images derived from biblical and folkloric sources to address the moral conflicts of his day. Hayes also monitors human behavior and evaluates its effects. In complex scenes packed with minute detail, he projects the absurdity of human interaction in a globalized world gone mad. His acid humor is reminiscent of Pieter Bruegel the Elder’s, only he takes on not just the country yokel but humanity at large.

Unlike many contemporary artists whose work often relies on theory and explanation, Hayes says little about his paintings, allowing the viewer to draw conclusions directly from his densely populated, multifocal, fictitious scenes and their hidden messages. “Far out to sea and west of Spain,/There is a country named Cokaygne,” goes the poetic description of medieval utopia that could be describing Hayes’ destinations, “No place on earth compares to this/For sheer delightfulness and bliss.” At first glance, his colorful paintings appear playful and lyrical, full of movement and intrigue. “There’s no fly or flea or louse/In clothes, in village, bed, or house;/There’s no thunder, sleet, or hail,/Or any nasty worm or snail.” But on closer inspection, a story unfolds that is often disturbing as much as captivating, dark as well as enlightening.

“They bury their Dead with their Heads directly downwards; because they hold an Opinion, that in eleven Thousand Moons they are all to rise again; in which Period, the Earth (which they conceive to be flat) will turn upside down, and by this Means they shall, at their Resurrection, be found ready standing on their Feet,” wrote Jonathan Swift about the inhabitants of Lilliput, in Gulliver’s Travels. Swift, continuing in the tradition of Herodotus, wrote about travel adventures. But, an inveterate satirist, he spiced them liberally with biting wit intended to upset and reform a malfunctioning society. “The chief end I propose to myself in all my labours is to vex the world rather than divert it.”

In The Russians knew perfectly well …, Hayes’ travel report seems to marry the wide-eyed astonishment of Herodotus with the edginess of Swift. Strange things happen in far off lands. But not even Hieronymus Bosch could have anticipated an angle as original and frightful as Hayes’. This time it is not the natives who demonstrate outlandish behavior but the visitors. Animals, he suggests, once lived happily in the wild, munching and frolicking in a potent state of anarchic freedom, living and dying their natural lives and deaths. Then humans arrived in their iron birds bringing their traps, their needs, their greed, their haplessness, and their neuroses.

The scene unfolds inside and outside the airplane and in some vacuous unreal landscape beneath. The panoramic view, a carnival of shape and color, yields a diminutive cosmos of stunning complexity. Animals, moved away from their natural habitat and become domesticated, have turned into caricatures of themselves, mindlessly engaged in meaningless tasks for no reason. The cartoonlike elephant on the upper left corner covers the eyes in dismay; the giraffe is clearly distressed. Awash in human fashions, the animals exhibit bizarre symptoms, biting themselves and each other or perched weirdly on floating vegetation.

Human behavior, in ancient Babylon, Lilliput, or Milikapiti, has cultural, economic, and public health consequences. Ecotourism has attracted people to remote animal habitats, and commerce has moved animals to new environments. Despite evidence of disease risks, demand for exotic pets is high. Despite inherent hazards (Buruli ulcer, malaria, dengue, avian flu, norovirus infection), humans move freely around the globe. “People,” Hayes says, “invariably find creative and elaborate ways of maintaining their perception, against all the available evidence, rather than questioning their perception of reality.”

In the wild, animals had no expectations. They did not travel far, nor did they carry luggage. Their happiness was guaranteed. Now, part and parcel of public transportation, they have lost not just their innocence and wildness but also the natural quarantine rendered by the borders of their habitat. And their bags are packed with more than human expectations. They have joined the growing zoonoses network, unknowingly moving microorganisms around the globe and expanding the scope and span of disease.
